# Iodine Deficiency in Pregnancy: The Effect on Neurodevelopment in the Child

**DOI:** 10.3390/nu3020265

**Published:** 2011-02-18

**Authors:** Sheila A. Skeaff

**Affiliations:** Department of Human Nutrition, University of Otago, PO Box 56, Dunedin 9054, New Zealand; Email: sheila.skeaff@otago.ac.nz; Tel.: +643-479-7944; Fax: +643-479-7958

**Keywords:** iodine, pregnancy, child, cretinism, neurodevelopment

## Abstract

Iodine is an integral part of the thyroid hormones, thyroxine (T_4_) and tri-iodothyronine (T_3_), necessary for normal growth and development. An adequate supply of cerebral T_3_, generated in the fetal brain from maternal free T_4_ (fT_4_), is needed by the fetus for thyroid hormone dependent neurodevelopment, which begins in the second half of the first trimester of pregnancy. Around the beginning of the second trimester the fetal thyroid also begins to produce hormones but the reserves of the fetal gland are low, thus maternal thyroid hormones contribute to total fetal thyroid hormone concentrations until birth. In order for pregnant women to produce enough thyroid hormones to meet both her own and her baby’s requirements, a 50% increase in iodine intake is recommended. A lack of iodine in the diet may result in the mother becoming iodine deficient, and subsequently the fetus. In iodine deficiency, hypothyroxinemia (*i.e.*, low maternal fT_4_) results in damage to the developing brain, which is further aggravated by hypothyroidism in the fetus. The most serious consequence of iodine deficiency is cretinism, characterised by profound mental retardation. There is unequivocal evidence that severe iodine deficiency in pregnancy impairs brain development in the child. However, only two intervention trials have assessed neurodevelopment in children of moderately iodine deficient mothers finding improved neurodevelopment in children of mothers supplemented earlier rather than later in pregnancy; both studies were not randomised and were uncontrolled. Thus, there is a need for well-designed trials to determine the effect of iodine supplementation in moderate to mildly iodine deficient pregnant women on neurodevelopment in the child.

## 1. Introduction

Despite considerable progress over the last 25 years, iodine deficiency is still one of the most common micronutrient deficiencies in the world today. Iodine deficiency results in a wide spectrum of adverse consequences throughout the lifecycle. Of greatest concern is the effect of iodine deficiency on the developing brain. The most serious effect of iodine deficiency is cretinism, which occurs in women who are severely iodine deficient during pregnancy. The promotion of iodised salt by the World Health Organization (WHO) and the International Council for the Control of Iodine Deficiency Disorders (ICCIDD) has helped to alleviate endemic cretinism in many parts of the world. Attention is now turning to the adverse effects of moderate and mild iodine deficiency in pregnancy. Although iodine deficiency in pregnancy will affect both the mother and the child, little attention has been paid to the consequences of iodine deficiency on maternal health. This review will describe the role iodine plays, via thyroid hormones, in the development of the brain from conception to birth and present evidence of the impact of severe, moderate, and mild iodine deficiency in pregnancy on neurodevelopment in the child.

## 2. The Role of Thyroid Hormones in Fetal Brain Development

Iodine is essential for the production of the thyroid hormones, thyroxine (T_4_) and the 3,5,3'-triiodothyronine (T_3_), which are vital for normal growth and development particularly of the brain and central nervous system. Maternal thyroid hormones are found in the embryonic cavities ~4 weeks after conception despite the placenta acting as a barrier designed to prevent excessively high levels of free T_4_ (fT_4_) and T_3_ from reaching the fetus before they are needed [1,2]. Thyroid hormones are not believed to play a role in very early fetal development as studies have shown that nuclear receptors for thyroid hormones are only present in the fetal brain from ~8–9 weeks gestation reaching adult levels by 18 weeks gestation [2]. Before midgestation, the mother is the only source of cerebral T_3_, which is generated in the fetal brain by type II 5'-iodothyronine deiodinase from maternal fT_4_. The first stage of thyroid hormone dependent neurodevelopment depends on an adequate supply of maternal fT_4_, and begins in the second half of the first trimester. This stage includes neuronal proliferation and the onset of neuronal migration in the cerebral cortex, hippocampus and medial ganglionic eminence, with the latter processes starting in the first trimester and continuing into the early part of the second trimester [2]. Towards the end of the first trimester, in response to high concentrations of placental human chorionic gonadotropin (hCG), there is a surge in maternal T_4_ and a decline in thyroid stimulating hormone (TSH), a mechanism thought to ensure that adequate fT_4_ is supplied to the fetus during this period [2,3]. Around the beginning of the second trimester the fetal thyroid begins to produce hormones, however, the full development of the pituitary-portal vascular system in the fetus does not occur until ~18–20 weeks gestation [1]. The second stage of thyroid hormone neurodevelopment includes neurogenesis, neuron migration, axonal growth, dendritic branching and synaptogenesis, glial cell differentiation and migration, and the onset of myelination [2]. The third stage occurs after birth. Although the concentration of T_4_ in the fetus increases as gestation progresses, the reserves of the fetal gland are low and the gland itself does not fully mature until birth, thus maternal thyroid hormones continue to contribute to total fetal thyroid hormone concentrations until birth. It is no surprise, therefore, that low fT_4_ occurs in premature babies as they are deprived of a maternal supply, and this may partly account for some of the developmental delay often apparent in such children [2].

A 50% increase in iodine intake is recommended in order for pregnant women to produce enough thyroid hormones to meet fetal requirements [4]. A lack of iodine in the diet may result in the mother becoming iodine deficient, and subsequently the fetus. The mother and the fetus, however, respond differently to this situation, with the mother remaining euthyroid and fetus becoming hypothyroid [3]. Iodine deficient pregnant women remain euthyroid for two reasons. Firstly, the increase in maternal fT_4_ that occurs at the end of the first trimester in response to hCG depresses the concentration of maternal TSH. Secondly, the maternal thyroid gland responds to a state of relative iodine deficiency by invoking the same responses in the thyroid gland as would occur in the non-pregnant state, such as increased iodine trapping, preferential synthesis of T_3_ over T_4_, hyperplasia, and eventually goitre. Thus, the woman will appear to be euthyroid as both her TSH and T_3_ concentration will fall within the normal reference range. In such situations, localised hypothyroxinemia occurring in specific parts of the developing fetal brain is believed to be responsible for the neurodevelopmental damage seen in iodine deficiency [5]. The autoregulatary mechanisms available to the mother do not take place in the fetus because the fetal gland has not fully matured. Consequently, in the fetus there is a decreased synthesis and secretion of T_4_ and T_3_, and an increase in the concentration of TSH, resulting in fetal hypothyroidism [3]. This explains why neonatal TSH is used as an index of iodine deficiency in a population. 

A number of studies have been undertaken in women living in areas of adequate iodine status who have low T_4 _concentrations in pregnancy, usually as a result of thyroid disease in the mother and not from iodine deficiency per se. Nonetheless, the assumption made by scientists working in this area is that, regardless of the cause, the functional consequences of abnormal thyroid hormones on neurodevelopment in the fetus are the same; however, this assumption is untested. In a study of Dutch women with fT_4_ < 10th percentile during pregnancy it was found that their children had poorer psychomotor development at 10 months of age [6], and again at one and two years of age [7] than children whose mothers had higher T_4_ levels in pregnancy. A more recent study found that pregnant Dutch women with fT_4_ < 10th percentile at 12 weeks gestation had infants with significantly lower orientation scores as assessed using the Neonatal Behavioural Assessment Scale than mothers with fT_4_ between the 50th and 90th percentiles [8]. Zoeller and Rovet discuss the role of thyroid hormones on brain development using data obtained from studies of maternal hypothyroidism and maternal hypothyroxinaemia, although as with the previous studies, these subjects were not iodine deficient [9]. They suggest that decreased thyroid hormones early in pregnancy may cause problems in visual attention, visual processing, and gross motor skills, while a lack of thyroid hormones later in pregnancy results in further problems with visual skills, slower processing speeds, and fine motor skills. Together, these observational studies provide evidence that a variation in maternal hormones influence normal brain development. Whether similar findings would occur in women with healthy thyroids but who are iodine deficient, needs further investigation. 

In summary, it is clear that low concentrations of maternal T_4_, in particular, can hinder the development of the fetal brain. If severe to moderate iodine deficiency is present, the damage to the developing brain in the first half of pregnancy is caused by hypothyroxinemia of a euthyroid mother, which can continue in the second half of pregnancy and is then further aggravated by hypothyroidism in the fetus. It is uncertain, however, if this occurs in situations of moderate or mild iodine deficiency resulting from low dietary intakes. 

## 3. Determining the Severity of Iodine Deficiency in Pregnancy

One of the problems in this area of research is the difficulty in determining the severity of iodine deficiency in pregnant women. The mostly commonly used index for assessing iodine status in a population is the median urinary iodine concentration (MUIC) as determined from a casual or spot urine sample. A MUIC > 100 µg/L is indicative of adequate iodine status in children, men and non-pregnant women, with a MUIC of 50–99 µg/L, 20–49 µg/L, and <20 µg/L indicating mild, moderate, and severe iodine deficiency, respectively [10]. In 2007, WHO recommended a MUIC > 150 µg/L be used in pregnancy, however, no cut-offs for the severity of iodine deficiency in pregnancy have been proposed [10]. Because of this, researchers usually assume that the severity of iodine deficiency in pregnant women will be similar to the severity of iodine deficiency observed in children and adults living in the same region. For example, if moderate iodine deficiency exists in children, then pregnant women living in that area are also considered to be moderately iodine deficient. This view has some justification because cretinism is observed in areas of severe iodine deficiency but does not occur in areas of moderate to mild iodine deficiency, hence, pregnant women living in such areas must have less severe iodine deficiency (*i.e.*, moderate to mild). Given the large variability in the iodine concentration of casual urine samples, the iodine status of an individual cannot be determined from a casual urine sample. In other words, a pregnant woman cannot be classified as iodine deficient based on her urinary iodine concentration [11]. 

Pregnant women can have their thyroid hormones measured, with TSH and fT_4_ typically used to define subclinical hypothyroidism (TSH > 97.5th percentile, fT_4_ in normal reference range), overt hypothyroidism (TSH > 97.5th percentile and fT_4_ < 2.5th percentile), and hypothyroxinemia (TSH in normal reference range, fT_4_ < 2.5th percentile) [12]. There is debate as to whether all pregnant women should be screened for abnormal thyroid hormone concentrations in the first trimester. Changes in thyroid hormones can be caused by a variety of factors such as thyroid disease, and is not specific to iodine deficiency. Indeed, WHO do not recommend the routine use of TSH, T_4_ and T_3_ for monitoring iodine status in the adult population as these tests are relatively insensitive in moderate to mild iodine deficiency; MUIC provides sufficient information to assess iodine status in these groups. However, because of the importance of maternal thyroid hormone concentrations for normal fetal brain development, the establishment of trimester specific international reference ranges for thyroid hormones in iodine sufficient and deficient pregnant women could be useful in identifying at-risk women.

## 4. The Effect of Severe Iodine Deficiency in Pregnancy on Neurodevelopment in the Child

Severe iodine deficiency exists when more than 30% of children have goitre, the population has a MUIC < 20 µg/L, and pregnant women living in the area give birth to cretins. Cretinism is always associated with a significant impairment in mental function and/or defects in hearing, speech, stance, gait, hypothyroidism and growth [13]. There are two types of cretinism, although the symptoms of both types can sometimes be seen in the same individual. The more common form of cretinism is neurological cretinism, characterised by mental retardation, deaf mutism, squint, spastic diplegia, and disorders of the stance and gait. The less common form of cretinism is called myxoedematous or hypothyroid cretinism and is characterised by less severe mental retardation, dwarfism, hypothyroidism, and a variety of other physical symptoms such as coarse and dry skin, a husky voice, and delayed sexual maturation. Normal IQ in a population is 100, while the IQ of cretins has been reported to be around 30 [14]. In areas with endemic cretinism, around 5–15% of non-cretinous children will have impaired mental function with an IQ of 50–69; these children are sometimes referred to as “sub-cretins” [13]. Although factors such as the presence of goitrogens in the diet, thyroid auto immunity, and interactions with other trace elements such as selenium have been postulated to have a role in the development and type of cretinism, the overarching reason for cretinism is severe iodine deficiency in the mother [15]. The importance of adequate dietary iodine in preventing cretinism was highlighted in the 1960s in a large trial of 165,000 people living in a part of Papua New Guinea with severe iodine deficiency and endemic cretinism [16]. In this study, it was found that an injection of iodised oil before conception or in early pregnancy reduced the incidence of cretinism and improved the motor and cognitive functions of children compared with those women who received the placebo. Another study undertaken in 1990 in a remote province in China with endemic cretinism examined the effect of iodised oil given during pregnancy on neurological outcomes. Children of mothers given iodine earlier in pregnancy had improved cognitive outcomes compared to mothers given iodine later in pregnancy at both two years [17] and again when children were school-aged [18]. A meta-analysis of Chinese studies reported a decrease of 8.7 IQ points in children born to mothers living in severely iodine deficient areas untreated in pregnancy compared to supplemented mothers also living in severely iodine deficient areas [19].

It is unequivocal that severe iodine deficiency in pregnancy results in significant impairment in cognition in the child. It has been suggested that cretinism is at the far end of a spectrum of effects that iodine deficiency can have on the central nervous system, and that varying degrees of intellectual impairment can occur across this spectrum concordant with the degree of iodine deficiency. This theory has yet to be substantiated.

## 5. The Effect of Moderate to Mild Iodine Deficiency in Pregnancy on Neurodevelopment in the Child

A number of clinical trials published between 1991 and 2002 in pregnant women living in regions with moderate deficiency are summarized in Table 1 [20,21,22,23,24,25,26,27]. Daily iodine supplements ranging from 100 to 300 µg of iodine were taken by women in their first or second trimester of pregnancy. The majority of the studies showed that iodine supplements were effective in alleviating aspects of iodine deficiency in supplemented women compared to those taking the placebo. With regard to these studies, iodine expert Dr. Michael Zimmermann wrote “…because none of the trials measured long-term clinical outcomes, such as maternal goiter or infant development, the potential adverse effects of mild-to-moderate iodine deficiency during pregnancy remain unclear” [28]. In 2009, two randomised trials were published investigating the effect of iodine supplementation in moderately iodine deficient pregnant women on neurodevelopment in their children. Berbel *et al.* recruited three groups of pregnant women living in Spain at different phases of gestation; the first group of women had T_4_ concentrations >20th percentile at recruitment (*i.e.*, >0.92 ng/dL at 4–6 weeks gestation), while the second and third groups of women had T_4_ concentrations <10th percentile (*i.e.*, <0.83 ng/dL) at 12–14 weeks gestation and near term, respectively. All three groups of women were supplemented with 200 µg of iodine until the end of lactation [26]. When the children were 18 months old, the development quotient of children in mothers supplemented in the first group (*i.e.*, 4–6 weeks) was significantly higher than that of children whose mothers received supplements from 12–14 weeks gestation and near term. A limitation of this study is the small numbers of children tested, with less than 20 children in each of the three groups. Furthermore, the women supplemented later in pregnancy or at term were specifically selected because they had low fT_4_ (*i.e.*, <10th percentile) in pregnancy, while the women supplemented earlier in pregnancy had a higher fT_4_ (*i.e.*, >20th percentile), thus a difference in fT_4_ rather than the iodine supplementation may account for the findings. A second Spanish study conducted in an area of moderate iodine deficiency (*i.e.*, UIC of pregnant women in this area was <100 µg/L) by Velasco *et al*. found that children of mothers supplemented with 300 µg of iodine in the first trimester had higher psychomotor development scores than children from mothers who did not start supplementation until the last month of pregnancy [27]. A limitation of this study was that children were tested at different ages in this study (5.5 months *vs*. 12.4 months). Finally, both studies were not randomized, double-blind, placebo-controlled trials, and although they suggest that neurodevelopment in the child may be adversely affected by moderate iodine deficiency, they are certainly not definitive.

**Table 1:** Studies investigating the effect of iodine supplementation in pregnancy.

Author (Year)	Country	*n*	Methods	Baseline UIC µg/L	Effect on child neurodevelopment
Romano *et al.* (1991) [21]	Italy	35	Women in first trimester randomised to 0 or iodine supplement (120–180 µg I/day).	37	Not assessed.
Pedersen *et al*. (1993) [22]	Denmark	54	Women at 17–18 weeks gestation randomised to 0 or 200 µg I/day.	55	Not assessed.
Glinoer *et al*. (1995) [23]	Belgium	120	Euthyroid women with signs of excessive thyroid stimulation randomised to 0 or 100 µg I/day.	36	Not assessed.
Liesenkotter *et al.* (1996) [20]	Germany	118	Women at 10–12 weeks gestation given 300 µg I/day *vs*. untreated women.	53	Not assessed.
Nohr and Laurberg (2000) [24]	Denmark	144	Retrospective allocation at term based on self-reported intake of supplements containing 150 µg I/day.	Not given	Not assessed.
Antonangeli *et al*. (2002) [25]	Italy	86	Healthy women 10–16 weeks gestation randomised to 50 or 200 µg I/day.	74	Not assessed.
Berbel *et al.* (2009) [26]	Spain	96	Women 4–6 weeks gestation with FT4 > 20th percentile (Group1) *vs*. women with FT4 < 10th percentile at 12–14 weeks (Group 2) or at 37–40 weeks (Group3) given 200 µg I/day until end of lactation.	75	Brunet-Lezine developmental quotient of children at 18 months was: 101.8 in Group 1 *vs*. 92.2 in Group 2 (*p* < 0.05) or 87.5 in Group 3 (*p* < 0.001).
Velasco *et al.* (2009) [27]	Spain	191	Women <10 weeks gestation (Group 1) *vs*. last month of pregnancy (Group 2) given 300 µg I/day until end of lactation.	87	Bayley Psychomotor Development Index of children was: 108.74 in Group 1 *vs*. 102.65 in Group 2 (*p* = 0.02).

UIC: urinary iodine concentration; TSH: Thyroid Stimulating Hormone; FT4: Free Thyroxine.

The consequences of milder types of iodine deficiency (*i.e*., MUIC 100–150 µg/L) in pregnancy have yet to be elucidated. In countries, such as Australia and New Zealand, which have seen the re-emergence of mild iodine deficiency in the last two decades, there appears to be no obvious signs of impaired neurodevelopment over this period with children meeting the usual developmental milestones at appropriate times. It is possible that in mild iodine deficiency adaptive mechanisms conserve iodine in the mother, such that the mother can supply the infant with sufficient thyroid hormones for normal brain development; the parasitic nature of the fetus in pregnancy is well known. To date, there are no published studies examining the effect of iodine supplementation in mildly iodine deficient pregnant women on neurodevelopment in children, however, by 2015 the results of ongoing randomised, placebo-controlled, intervention trials being conducted in Thailand and India [29] should be available. Conducting such trials will become increasingly difficult in the coming years for a number of reasons: firstly, the promotion of strategies to improve iodine status will result in more populations becoming iodine sufficient around the world and less populations with iodine deficiency; and secondly, giving a placebo with no iodine to a group of women with iodine deficiency has been suggested to be unethical. This view is premature given the paucity of evidence and counters the philosophy of evidence-based health care.

## 6. Conclusion

Severe iodine deficiency during pregnancy is a known cause of cretinism and mental retardation. At present, there are limited data on the cognitive deficits of children born to mothers who are moderately iodine deficient in pregnancy. There is a need for well designed clinical trials investigating whether routine iodine supplementation of pregnant women living in regions of mild iodine deficiency improves neurodevelopment in children. 
